# Juglone Suppresses Inflammation and Oxidative Stress in Colitis Mice

**DOI:** 10.3389/fimmu.2021.674341

**Published:** 2021-08-05

**Authors:** Shuai Chen, Xin Wu, Zengli Yu

**Affiliations:** ^1^School of Public Health, Xinxiang Medical University, Xinxiang, China; ^2^Key Laboratory of Agro-ecological Processes in Subtropical Region, Institute of Subtropical Agriculture, Chinese Academy of Sciences, Changsha, China; ^3^School of Public Health, Zhengzhou University, Zhengzhou, China

**Keywords:** polyphenol, juglone, mice, DSS, colitis, inflammation, oxidation, microbiota

## Abstract

Juglone (JUG), a natural product found in walnut trees and other plants, shows potent antioxidant, antimicrobial, and immunoregulatory activities. However, it remains unknown whether JUG can alleviate ulcerative colitis. This study aims to explore the effect of JUG on dextran sulfate sodium (DSS)-induced colitis in mice. The mice were randomly assigned into three groups: the vehicle group, the DSS group, and the JUG group. The experiments lasted for 17 days; during the experiment, all mice received dimethyl sulfoxide (DMSO, 0.03% v/v)-containing water, while the mice in the JUG group received DMSO-containing water supplemented with JUG (0.04 w/v). Colitis was induced by administering DSS (3% w/v) orally for 10 consecutive days. The results showed that the JUG treatment significantly ameliorated body weight loss and disease activity index and improved the survival probability, colon length, and tissue damage. JUG reversed the DSS-induced up-regulation of proinflammatory cytokines, including interleukin (IL)-6, 12, 21, and 23, and tumor necrosis factor-alpha, and anti-inflammatory cytokines, such as IL-10 and transforming growth factor-beta, in the serum of the colitis mice. Additionally, the activation of mitochondrial uncoupling protein 2 and phospho-Nuclear Factor-kappa B p65 and the inhibition of the kelch-like ECH-associated protein 1 and NF-E2-related factor 2 induced by DSS were also reversed under JUG administration. Although the JUG group possessed a similar microbial community structure as the DSS group, JUG enriched potential beneficial microbes such as *Lachnospiraceae_NK4A136_group* but not pathogens such as Escherichia Shigella, which was dominative in DSS group, at the genus level. In conclusion, our results demonstrated that JUG could be a promising agent for UC prevention to regulate inflammatory cytokines and oxidative stress.

## Introduction

Ulcerative colitis (UC), one of the subtypes of inflammatory bowel disease (IBD), is highly prevalent in North America (0.29% in the USA) and Europe (0.51% in Norway), and it has shown an increasing prevalence in Asia, Africa, and South America (1). UC is a relapsing chronic and rectal intestinal disorder with superficial mucosal inflammation that could damage the inner intestinal wall, further leading to ulcerations, bleeding, and abdominal cramps, and pain (2). Although UC pathogenesis remains poorly understood, various lines of evidence have demonstrated that intestinal disorders induce immune-mediated inflammation, pathogen invasions, and inflammatory cell infiltration (2), which could further result in continues oxidative stress, inflammation, gut barrier damage, as well as, gut microbiota dysbiosis (3, 4).

Numerous polyphenols that have been reported to inhibit colitis in rodents and humans rely on their antioxidant, cell signaling pathways regulation, anti-inflammation, and anti-pathogen properties (5). Juglone (JUG) is a natural product found in the fruit husk, roots, bark, and leaves of walnut trees and other plants. JUG shows strong antioxidant properties, such as quenching of ROS, enzyme inhibition, and translation metal ion chelation. And JUG is potent in treating oxidative stress-associated diseases such as Alzheimer’s disease, kidney fibrogenesis, and liver fibrogenesis (6). JUG also has anticancer, antimicrobial, and immunoregulatory activities, and it can regulate energy metabolism and cell signaling pathways, such as inhibiting the protein kinase B pathway in prostate cancer cells (6–9). JUG has demonstrated antibacterial activity against pathogens such as *Staphylococcus aureus*, *Candida albicans*, and *Helicobacter pylori* (10, 11). Wang et al. reported that JUG inhibited tumor growth and metastasis, myeloid-derived suppressor cell accumulation, and interleukin (IL)-1β, IL-6, and tumor necrosis factor (TNF)-α production in colorectal cancer mouse models (12). Thus, we suspect JUG might alleviate colitis. However, little is known about the therapeutic effect and underlying mechanisms of JUG on UC.

This study aims to investigate the effects of JUG on dextran sulfate sodium salt (DSS)-induced colitis in mice. We hypothesize that JUG could inhibit inflammation and oxidative stress and regulate gut microbiota in colitis mice.

## Materials and Methods

### Animals and Experimental Design

**Figure 1:**
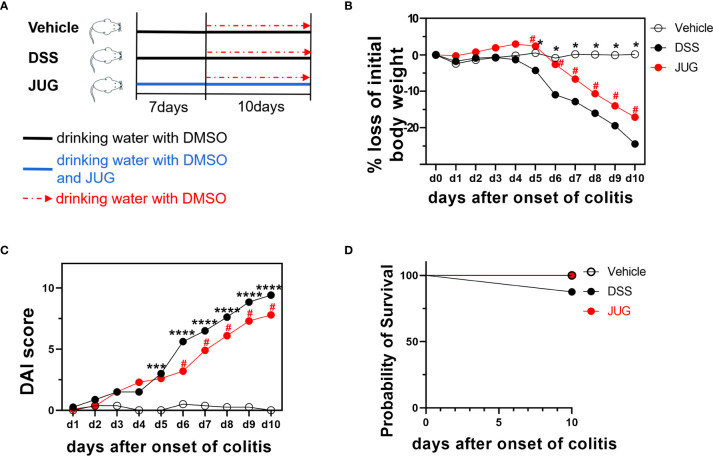
Bodyweight loss, the DAI score, and the survival probability of mice. **(A)** Schematic showing the treatment of each group. Bodyweight loss percentage **(B)**, DAI score **(C)**, and survival probability **(D)** during the DSS administration. Two-way ANOVA was used to analyze the difference between the DSS group and the vehicle/JUG group. *P < 0.05, ***P < 0.001, ****P < 0.0001, ^#^ *P* < 0.05.

The C57BL6/J mice (male, 6-8 week-old) were assigned into three groups using a randomized block design by body weight (n=8/group): the vehicle group (control group), the DSS group, and the JUG group. The experiment was continued for 17 days. All of the mice received drinking water containing 0.03% dimethyl sulfoxide (DMSO) (Aladdin, Shanghai, China) during the experiment. The mice of the JUG group received DMSO-containing water supplemented with 0.04 mg/ml of JUG (Aladdin, Shanghai, China) for 17 days. Colitis induction was performed by administering 3% (w/v) DSS (MP Biomedicals, MW 36.000–50.000) in tap water ad libitum for mice in the DSS and JUG groups from day 7 to day 17. The mice in the control group drank regular water (**Figure 1A**).

Bodyweight loss, stool consistency, bloody stool, and survival were recorded during the induction of colitis according to a standard scoring system (13), as shown in **Supplementary Table 1**. The sum of scores on weight loss, stool consistency, and bloody stool was calculated and recorded as disease activity index (DAI). Investigators were blinded to the group allocation while collecting data.

On the day 17, the mice were euthanized to collect the colon and colonic content following blood collection. About 0.5 cm length of colonic tissue was separated and fixed in 10% buffered formalin following colonic length measurement, and then the rest of colonic tissue was divided triplicately, snap-frozen in liquid nitrogen, and stored at -80°C before further processing. The blood was centrifuged at 3000 rpm for 10 minutes for serum separation.

### Histological and Immunohistochemical Analysis

The colon was dehydrated and embedded in paraffin wax after being fixed in formalin overnight. After being implanted, several sections of 5 um thickness were sliced according to standard methods (14). The sections were used for hematoxylin and eosin staining (H&E) and mitochondrial uncoupling protein 2 (Ucp1), nuclear factor erythroid 2-related factor 2 (Nrf2), and kelch-like ECH-associated protein (Keap) 1 immunohistochemical analysis. The histopathological damage of colon tissue was examined on light microscopy (Olympus, Tokyo, Japan) (× 200) by Image J software (National Institutes of Health, Bethesda, MD, USA). Histopathological index (HI) was assessed based on a semi-quantitative score criterion (**Supplementary Table 2**), which is the sum of scores on lymph node numbers, ulcerative area, epithelial changes, and inflammatory cell infiltrate.

Tissue sections used for immunohistochemical analysis were deparaffinized and hydrated. Then, the sections were retrieved in the citric acid buffer (pH 6.0) in a microwave oven. Furthermore, the tissue slide was treated with 3% H2O2 for 25 min and incubated with primary antibodies against UCP1, Nrf2, Keap1 (1:100; Servicebio Technology Co. Ltd., Wuhan, China) at 4°C overnight. After washing out the excess primary antibody, the samples were treated with Alex Fluor 647 conjugated secondary antibody (Cell Signaling Technology, Boston, MA) and DAPI (Thermo Scientific, Rockford, IL) for 1 h in the dark. The fluorescence was detected by a laser confocal microscope (Carl Zeiss, Germany).

### Antioxidant System Analysis in the Serum

The serum levels of Malondialdehyde (MDA), glutathione peroxidase (GSH-Px), and total anti-oxidative capacity (T-AOC) were analyzed, respectively, using assay kits following the manufacturer’s instructions (Beyotime Biotechnology, Shanghai, China).

### Enzyme-Linked Immunosorbent Assay (ELISA) Assays

The content of IL-6, 12, 21, 23, TNF-α, IL-10, and TGF-β in the serum was quantified by commercial ELISA kits (Multi Sciences Biotech, Hangzhou, China) according to the instruction of the manufacturer. The BioTek MQX200 microplate reader (BioTek Instruments Inc., Winooski, VT, USA) was used to analyze the optical density of colorimetric reaction at 450 nm. The standard curve was processed according to the optical density and the concentration of the standers using the mELISA software (QINMS, Guilin, China).

### Western Blot (WB) Assay

WB was performed following our previous protocol (15). The colon was homogenized in RIPA lysis buffer containing a complete protease and phosphatase inhibitor (Thermo Fisher Scientific, Shanghai, China) and centrifuged at 4°C for 20 min. The supernatant was collected to analyze the total protein concentration using a BCA protein assay kit (Beyotime Biotechnology, China). Equal amounts of proteins of each sample were separated on 10% SDS-PAGE and then transferred to PVDF membrane (Millipore; Billerica, MA, USA). The PVDF membrane was then incubated with specific primary antibodies (Ucp2,p65,p-p65, Nrf2, Keap1, and β-actin; Cell Signaling Technology, Danvers, MA, USA) at 4°C overnight following incubation with 5% skim milk for 2 h at room temperature. Subsequently, membranes were washed in TBST triply and incubated with HRP-conjugated secondary antibodies for 1 h at room temperature. An enhanced chemiluminescence kit (Tanon, Nanjing, China) was used to visualize the band. Carestream Molecular Imaging system (Carestream Health, Inc., USA) was used to analyze and quantify the intensity of protein bands, further to calculate the integrated optical density (IOD).

### 16S rRNA Gene Microbiome Analysis

According to our previous study (16), the Qiagen QIAamp DNA Stool Mini kit (Qiagen, Beijing, China) was used to extract the total genomic DNA of colonic contents. DNA was quantified by a Nanodrop spectrophotometer (Thermo Scientific, New York, USA), and its purity was tested on 1% agarose gel. Qualified samples were subsequently amplified the V3-V4 region of ribosomal DNA gene using the universal primers 338F (5’- ACTCCTACGGGAGGCAGCA-3’) and 806R (5’- GGACTACHVGGGTWTCTAA T-3’) and were then sequenced on an Illumina Miseq platform. Operational taxonomic units (OTUs) clustering was identified at 97% sequence identity. The Quantitative Insights into Microbial Ecology (QIIME) software was used to perform alpha-diversity, Venn analysis, unweighted UniFrac principal coordinate analysis (PCoA), weighted unifrac PCoA, the linear discriminant analysis (LDA) effect size (LEfSe) algorithm, and PICRUSt2 algorithm. All diagram was processed by using R software (V2.15.3).

### Statistical Analysis

GraphPad Prism 9 (GraphPad Software, California, United States) was used to perform the statistical analysis. The two-way ANOVA or mixed model methods were used to analyze the difference of bodyweight loss and DAI data between the Vehicle or JUG group and the DSS group. The unpaired t-test or Wilcoxon signed-rank test was used for statistical analysis of other data. The results were presented as mean ± standard error of the mean (SEM), and *P-value <*0.05 was considered statistically significant.

## Results

### JUG Attenuated DSS-Induced Colitis

**Figure 2:**
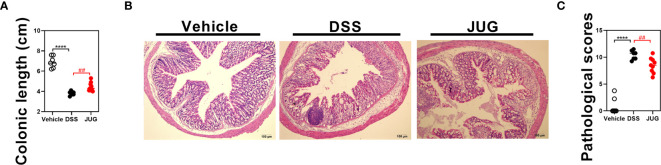
Colon length and pathological scores of mice. **(A)** Colon length of each mouse in each group. **(B)** Representative H&E staining images of the colon (×100) and **(C)** pathological scores from the colonic sections in each group. ****P < 0.0001, ^##^ *P* < 0.01.

The DAI and pathological scores were monitored in colitis mice to assess the role of JUG in colitis. Water supplemented with JUG significantly reversed DSS-induced (*P*< 0.05) weight loss from day 5 (**Figure 1B**) and also the DAI score increase from day 7 (**Figure 1C**). However, the probability of survival was similar between the DSS group and the vehicle or JUG group (**Figure 1D**). JUG also ameliorated (*P*< 0.05) colon shortening (**Figure 2A**) and reduced (*P*< 0.05) the pathological score (*P*< 0.05) (**Figures 2B, C**) in colitis mice. These data indicated that JUG ameliorated DSS-induced colitis.

### JUG Inhibited DSS-Induced Inflammation

**Figure 3:**
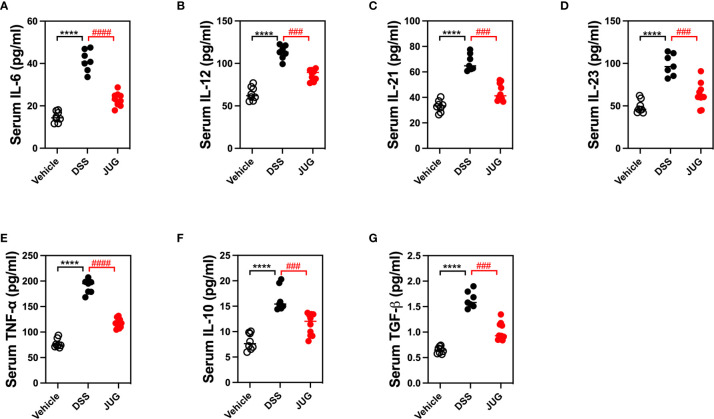
ELISA analysis to test DSS-induced inflammation. ELISA was used to analyze serum cytokines, including IL-6 **(A)**, 12 **(B)**, 21 **(C)**, 23 **(D)**, TNF-α **(E)**, IL-10 **(F)**, and TGF-β **(G)**. ****P < 0.0001, ^###^ *P* < 0.001, ^####^ *P* < 0.0001.

Serum proinflammatory cytokines were evaluated to test the effects of JUG on DSS-induced inflammation. JUG reversed (*P*< 0.05) the enrichment of IL-6, 12, 21, and 23, and TNF-α induced by DSS (**Figures 3A–E**), indicating that JUG reversed DSS-induced inflammation. However, the anti-inflammatory cytokines (IL-10 and TGF-β) were also increased in the DSS group, which was also reduced by JUG administration (**Figures 3F, G**). These results indicated that JUG reversed DSS-induced inflammation.

### JUG Mitigated DSS-Induced Oxidative

**Figure 4:**
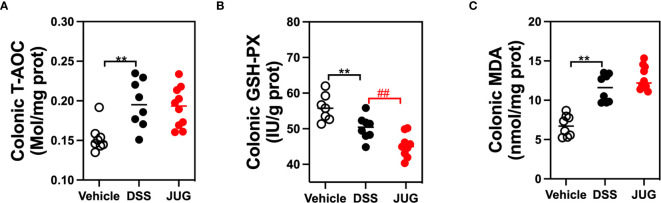
Immunohistochemical examination for analysis of the DSS-induced oxidative damage. The colonic T-AOC **(A)**, MDA **(B)**, and GSH-Px **(C)** were analyzed by immunohistochemical examination. **P < 0.01, ^##^ *P* < 0.01.

**Figure 5:**
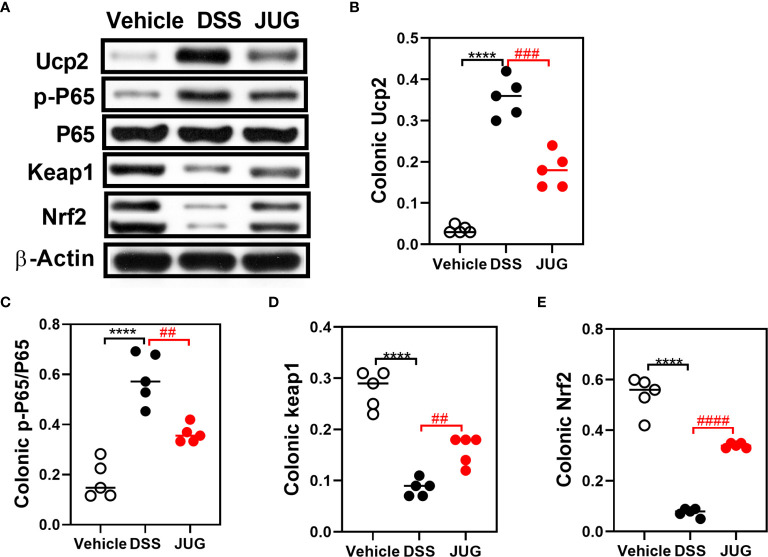
WB for analysis of the DSS-induced oxidative damage. Colonic p-P65, P65, Keap1 Ucp2, and Nrf2 expression on the protein level were analyzed by WB. **(A)** Representative western blot images of the colonic p-P65, P65, Keap1, Ucp2, NRF2, and β-Actin proteins. **(B–E)** Relative densitometric of Ucp2, p-P65/P65, Keap1, and Nrf2 in the colon. ****P < 0.0001, ^##^ *P* < 0.01, ^###^ *P* < 0.001, ^####^ *P* < 0.0001.

**Figure 6:**
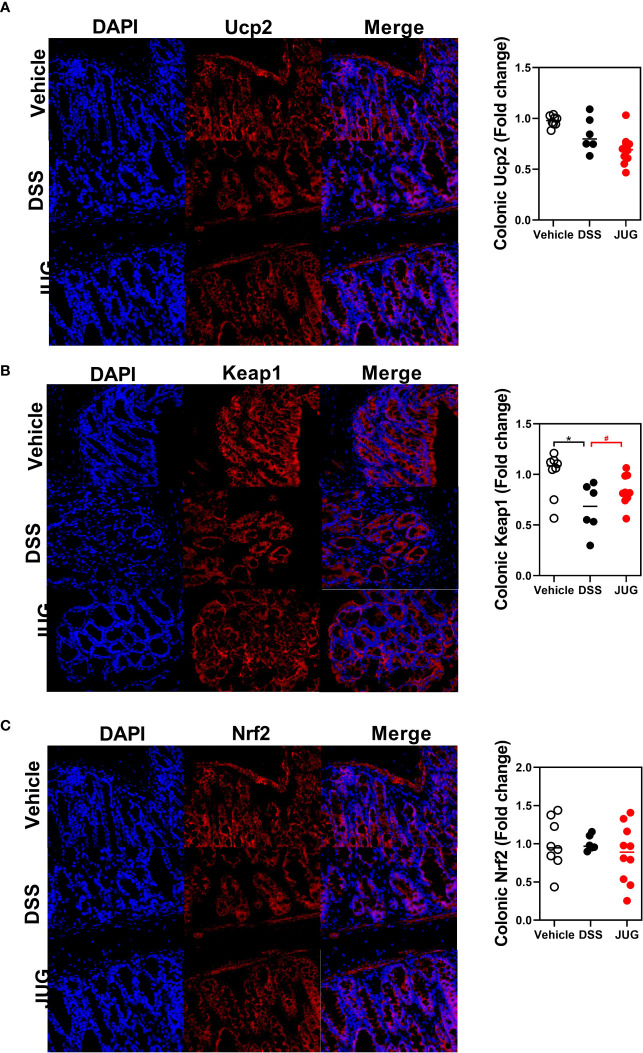
Immunofluorescence staining for analysis of the DSS-induced oxidative damage. Immunofluorescence staining was assigned to measure the colonic expression of Ucp2 **(A)**, Keap1 **(B)**, and Nrf2 **(C)**. * meant *P* < 0.05, and ^#^ meant 0.1 < *P* < 0.05, vs. the DSS group.

The effects of JUG on intestinal oxidative stress were measured by detection of the colonic T-AOC, GSH-Px, and MD. The results showed that colonic T-AOC and MDA were increased and were not affected by JUG (**Figure 4**). GSH-Px was lower in the DSS group than that of the controls, and JUG also decreased the GSH-Px concentration (**Figures 4B**). We next evaluated the colonic expression of p-p65, p65, Keap1, Ucp2, and Nrf2 using WB (**Figure 5A**) and found that JUG reversed the effects of DSS on activating Ucp2 and p-p65 and inhibiting Keap1 and Nrf2 (**Figures 5B–E**). Immunofluorescence staining was also performed to measure the colonic expression of Ucp2, Keap1, and Nrf2 (**Figure 5**). The IOD of Ucp2 and Nrf2 were similar between the DSS group and the vehicle or JUG group (**Figures 6A, C**). The Keap 1 expression agreed with the result of WB (**Figure 6B**).

### JUG Had Limited Effects on the Gut Microbiota of Colitis Mice

**Figure 7:**
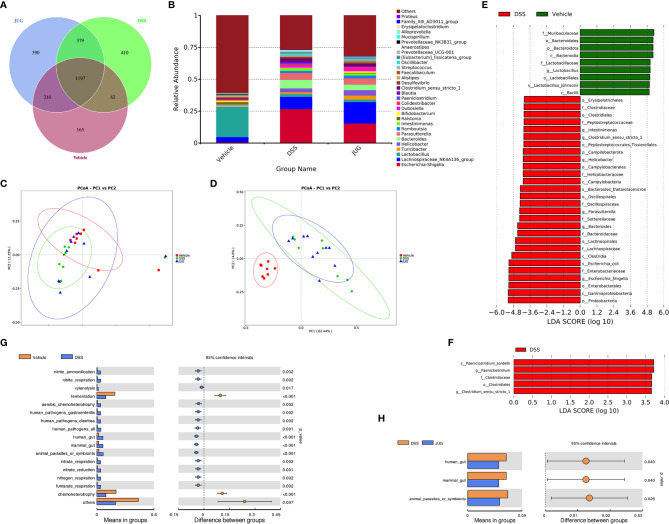
16S rRNA sequencing for DSS-induced gut microbial detection. 16S rRNA sequencing was employed to measure the impact of JUG on the gut microbiota. **(A)** The Venn graph of the three groups; **(B)** the relative abundance of the microbial population at the genus level, the unweighted unifrac PCoA diagram. **(C)** The weighted unifrac PCoA diagram of each group. **(E, F)** The LDA score of the LEfSe analysis between the DSS and Vehicle or JUG group, and the different gut microbiota functions between the DSS and vehicle LDA score > 4 **(G)** or JUG group LDA score > 3.6 **(H)**.

1197 core OTUs of a total of 2813 OTUs were shared among the three groups, while the unique OUTs in the vehicle group, the DSS group, and the JUG group were 165, 410, and 390, respectively (**Figure 7A**). *Lactobacilllus* and *Lachnospiraceae_NK4A136_group* were dominant in the vehicle group, while *Escherichia-shigella* and *Lachnospiraceae_NK4A136_group* were dominant in the DSS and JUG groups, separately, at the genus level (**Figure 7B**). The alpha-diversity analysis and PcoA showed no difference between the vehicle or the JUG group and the DSS group (**Table 1**, **Figure 7C**). The weighted unifrac PCoA demonstrated that the vehicle group was separated from the DSS group (**Figure 7D**). The LEfSe analysis indicated that *Lactobacillus* and *L. johnsonii* were enriched in the vehicle group, while *Escherichia Shigella, Escherichia coli, Bacteroides, B. thetaiotaomicron, Clostridium sensu_stricto_1, Parasutterella*, and *Helicobacter* were enriched in colitis mice, could be used as biomarkers of DSS-induced colitis (LDA score>4, **Figure 7E**). In addition, We found *Paeniclostridium, P. sordelllii*, and *Clostridium sensu_stricto_1* were less enriched in colitis mice admistered by JUG (LDA score > 3.6, **Figure 7F**). JUG reduced the DSS-activated function of the mammal gut and animal parasites or symbionts (**Figures 7G, H**). These results demonstrated that JUG had limited effects on DSS-induced gut microbiota shifts.

**Table 1:** Alpha diversity indexes of each group.

	Vehicle	DSS	JUG	SEM
Shannon	5.1379	5.2039	5.63	0.4857
Simpson	0.9134	0.84	0.9252	0.0617
Chao1	616.808	765.3958	751.7953	68.0811
ACE	632.8906	785.9117	770.2408	69.8313

The community richness estimator, including the Chao1 estimator and the ACE estimator, and the community diversity indexes, including the Shannon index and the Simpson index, were evaluated. An unpaired t-test was used for the analysis between the vehicle/JUG group and the DSS group.

## Discussion

The pathogenesis of colitis, a globally prevalent disease that risks the health of millions of people, is still not clearly elucidated. New efficiency bioactivators for managing UC still need to be investigated (17). This study found that JUG potently alleviated DSS-induced colitis as indicated by suppressed proinflammatory cytokines and oxidative stress. Additionally, JUG significantly reversed the DSS-induced activation of Ucp2 and p-p65 and the inhibition of Nrf2 signaling. These data may provide new insights into the mechanisms of JUG for controlling symptoms of UC.

Cytokines and cytokines networks are critical regulators in intestinal cellular interactions to modify the gut epithelial barrier function, intestinal immunity and defense, and tissue repair to maintain gut health (18). Proinflammatory cytokines, such as IL-1β, TNF-α, and IL-6 driven by intestinal mononuclear phagocytes or epithelial cells, are the inflammatory effectors that initiate intestinal inflammation in UC (19); while IL-12 and IL-23 are regarded as drivers of the pathogenic response to further promote intestinal inflammation (19). Anti-inflammatory cytokines, such as IL-10 and TGF-β induced by microbial or metabolites, show vital regulatory roles in immunity tolerance in intestinal inflammation (18). This study found that JUG prevented the elevation of proinflammatory cytokines, including IL-1β, IL-6, IL-12, IL-23, and TNF-α, induced by DSS treatment. Consistent with our findings, JUG suppressed TNF-α, IL-1β, and IL-6 expression in high-fat diet rats (20). JUG was also reported to inhibit TNF‐α and Nuclear Factor-kappa B (NF-κB) production in colonic cancer cells (21). However, there is controversy because DSS enhanced serum IL-10 and TGF-β levels while JUG reduced serum levels. As a typical anti-inflammatory cytokine produced by various cells (i.e., B and T lymphocytes, macrophages, and dendritic cells) in IBD, IL-10 plays a central role in gut homeostasis and colitis prevention (22). Although the IL-10 or IL-10 receptor deficiency mice showed severe gut inflammation in IBD (23), previous studies regarding serum IL-10 in IBD showed inconsistent results; as stated, it decreased, increased, or a similar serum IL-10 was observed in IBD patients (22, 24). Additionally, IL-10 supplementation did not achieve the desired effect, and an efficient acceptable IL-10 target therapy is still lacking in humans (25). IL-10 treatment was only efficient in initial colitis but not in established IBD of the mouse model (25). Thus, the role of IL-10 in this study is not clear and requires further study. Moreover, TGF-β deficiency induces colitis in mice; however, it also shows proinflammatory properties locally of TGF-b in IBD (26, 27). Consistent with our results, Zhao et al. demonstrated that TGF-β1 contents increased in the serum and colon of DSS treated rats, while honey, honey polyphenol, and sulfasalazine administration decreased the TGF-β1 in both the plasma and colon (26). These results indicated that preserving the cytokines at normal levels may ascribe the effect of JUG for attenuating UC symptoms and further reconstructing the intestinal barrier.

Excess reactive oxygen production is usually observed in chronic intestinal inflammation, indicating that oxidative stress plays a vital role in IBD. Oxidative stress and inflammation facilitate each other, further impairing the structure and function of the intestine at the active stage of colitis (28, 29). In UC, NF-κB pathways were reported significantly activated, resulting in the expression of proinflammatory cytokines. Nrf2 could respond to oxidative stress by modulating cytoprotective gene expressions (30, 31). This study found that JUG inhibited Ucp2 and p-p65 while activated Nrf2 in colitis. Accumulating evidence has shown that JUG exerts beneficial effects under inflammatory situations *via* NF-κB pathway suppression (20). What’s more, 2-methoxy-7-acetonyljuglone, a juglone derivative, was also found to possibly increase the nuclear localization of Nrf2 and its target genes (32). Therefore, the beneficial effect of JUG against colitis may be attributed, at least partially, to the reconstitution of the Nrf2-mediated cellular anti-oxidative system and the suppression of NF-κB induced inflammation.

Dysbiosis of intestinal flora, commonly characterized by an overgrowth of pathogenic bacteria and a decrease in beneficial bacteria, leads to an improved mucosal immune response and, consequently, inflammation. The Lefse analysis indicated that DSS enriched pathogenic bacteria, such as *Clostridium_sensu_stricto_1*, and *Escherichia Shigella*, which were less enriched under JUG administration. *Escherichia Shigella* was the dominative member on the genus level in the DSS group, while *Lachnospiraceae_NK4A136_group* genus level was dominant in the JUG-treated colitis mice. The *Lachnospiraceae_NK4A136_group* has been found to be beneficial to gut health and is regarded as an anti-inflammatory factor due to its production of short-chain fatty acids (33). Similar to our results, Dou et al. reported that sodium butyrate treatment improved colitis and increased the *Lachnospiraceae_NK4A136_group* in DSS-induced colitis mice (34). Hence, improvements of the *Lachnospiraceae_NK4A136_group* and reductions in *Escherichia-Shigella* may partially benefit the restoration of gut microbiota and the reestablishment of intestinal epithelium after JUG treatment.

## Conclusions

In summary, JUG alleviated DSS-induced colitis against colonic inflammation and oxidative stress in DSS-treated mice. The underlying mechanisms of JUG preventing colitis involved the activation of the Nrf2 and the inhibition of NF-κB signaling pathways. Moreover, the effects of JUG might also associated with the regulating effect on gut microbiota such as *Lachnospiraceae_NK4A136_group* and *Escherichia Shigella*. These findings enhance our understanding of the mechanisms of JUG on colitis.

## Data Availability Statement

The original contributions presented in the study are included in the article/**Supplementary Material**. Further inquiries can be directed to the corresponding author.

## Ethics Statement

The animal study was reviewed and approved by the Animal Welfare Committee of the Institute of Subtropical Agriculture, Chinese Academy of Sciences.

## Author Contributions

SC and ZY designed the experiment. SC and XW performed the experiment, analyzed the data, and prepared tables and figures. SC and ZY prepared the manuscript. All authors contributed to the article and approved the submitted version.

## Funding

This research was supported by Zhongyuan Science and Technology Innovation Leadership Program No.214200510016.

## Conflict of Interest

The authors declare that the research was conducted in the absence of any commercial or financial relationships that could be construed as a potential conflict of interest.

## Publisher’s Note

All claims expressed in this article are solely those of the authors and do not necessarily represent those of their affiliated organizations, or those of the publisher, the editors and the reviewers. Any product that may be evaluated in this article, or claim that may be made by its manufacturer, is not guaranteed or endorsed by the publisher.

## Supplementary Material

Supplementary Table 1 DAI score protocol. DAI score was used to evaluate colitis’s clinical activity, and it was the total sum of body weight loss score, stool consistency score, and bloody stools score.

Supplementary Table 2 Pathological score protocol. The pathological score was used to evaluate intestinal inflammation, and it was the total sum scores of lymph node numbers, ulcerative area (%), epithelial changes, and inflammatory cell infiltrate.
