# Supplementary material for: Juglone Suppresses Inflammation and Oxidative Stress in Colitis Mice
Source: Front Immunol. 2021 Aug 5;12:674341. doi: 10.3389/fimmu.2021.674341 (PMC8375437; doi:10.3389/fimmu.2021.674341)
Supplement: Supplementary Table 1 — DAI score protocol. DAI score was used to evaluate colitis’s clinical activity, and it was the total sum of body weight loss score, stool consistency score, and bloody stools score. [file Table_1.docx]

**Supplementary Table 1. DAI score protocol**

| **Score** | **Bodyweight loss (%)** | **Stool consistency** | **Bloody stools** |
| --- | --- | --- | --- |
| 0 | < 5 | normal stool | normal |
| 1 | 5 ~ 10 | soft stool | dark or dark red colored |
| 2 | 10 ~ 15 | mucoid stool | with traces of blood |
| 3 | 15 ~ 20 | watery stool | visible rectal bleeding |
| 4 | > 20 |  |  |

DAI score was used to evaluate colitis's clinical activity, and it was the total sum of body weight loss score, stool consistency score, and bloody stools score.
