# Supplementary material for: Juglone Suppresses Inflammation and Oxidative Stress in Colitis Mice
Source: Front Immunol. 2021 Aug 5;12:674341. doi: 10.3389/fimmu.2021.674341 (PMC8375437; doi:10.3389/fimmu.2021.674341)
Supplement: Supplementary Table 2 — Pathological score protocol. The pathological score was used to evaluate intestinal inflammation, and it was the total sum scores of lymph node numbers, ulcerative area (%), epithelial changes, and inflammatory cell infiltrate. [file Table_2.docx]

**Supplementary Table 2. Pathological score protocol**

| **Score** | **Lymph node numbers** | **Ulcerative area (%)** | **Epithelial changes** | **Inflammatory cell infiltrate** |
| --- | --- | --- | --- | --- |
| 0 | 0 | normal | normal | normal |
| 1 | 1 | <25% | goblet cell loss | minimal scattered infiltrate in mucosa |
| 2 | 2 | 25 ~ 50 | severe goblet cell loss | mild dense infiltrate in mucosa and submucosa |
| 3 | 3 | 50 ~ 75 | crypt abscesses | moderate scattered in submucosa |
| 4 | >= 4 | >75 | severe crypt abscesses | marked dense infiltrate in mucosa and submucosa |

The pathological score was used to evaluate intestinal inflammation, and it was the total sum scores of lymph node numbers, ulcerative area (%), epithelial changes, and inflammatory cell infiltrate.
